# Body mass index affects imatinib exposure: Real‐world evidence from TDM with adaptive dosing

**DOI:** 10.1111/fcp.13049

**Published:** 2025-01-03

**Authors:** Paul Maroselli, Raphaelle Fanciullino, Julien Colle, Laure Farnault, Pauline Roche, Geoffroy Venton, Régis Costello, Joseph Ciccolini

**Affiliations:** ^1^ PRISM Biogénopôle La Timone University Hospital of Marseille, APHM Marseille France; ^2^ COMPO Centre de Recherche en Cancérologie de Marseille Inserm U1068 Marseille France; ^3^ Pharmacy Unit La Conception University Hospital of Marseille APHM Marseille France; ^4^ Hematology Dept La Conception University Hospital of Marseille APHM Marseille France

**Keywords:** adaptive dosing, body mass index, Imatinib, pharmacokinetics, therapeutic drug monitoring

## Abstract

**Background:**

Imatinib is the treatment of elderly or frail patients with chronic myeloid leukemia (CML). Trough levels of around 1000 ng/ml are considered as the target exposure.

**Objectives:**

We searched for baseline parameters associated with imatinib pharmacokinetics, and studied the clinical impact of subsequent adaptive dosing.

**Methods:**

We present data from 60 adult CML patients upon imatinib with therapeutic drug monitoring (TDM) and adaptive dosing.

**Results:**

Mean trough levels after treatment initiation were 994.2 ± 560.6 ng/ml with 56% inter‐patient variability). Only 29% of patients were in the therapeutic range. Body weight, height, body surface area, body mass index (BMI), and age were associated with imatinib plasma levels on univariate analysis. Age and BMI remained the only parameters associated with imatinib trough levels on multivariate analysis. As severe toxicities have been previously reported in patients with low BMI treated with standard imatinib, we evaluated the extent to which low BMI may lead to plasma overexposure. We found a statistically significant difference in trough imatinib levels in patients with BMI < 18.5 kg/m^2^, with exposure +61.5% higher than in patients with 18.5 < BMI ≤  24.9 and +76.3% higher than in patients with BMI ≥ 25. After TDM with adaptive dosing, a statistically significant difference in dosing between patients was observed, with doses ranging from 200 to 700 mg. No difference in toxicity or efficacy was observed regardless of BMI after adaptive dosing.

**Conclusion:**

Our data suggest that low BMI has a significant impact on imatinib exposure but that pharmacokinetically‐guided dosing limits its clinical impact in patients.

## 1 INTRODUCTION

Imatinib (Gleevec®), a selective BCR‐ABL1 kinase inhibitor, was the first approved oral targeted therapy and a game changer in the treatment of chronic myeloid leukemia (CML) and, to a lesser extent, gastrointestinal stromal tumor (GIST) [1]. Although now superseded by newer targeted therapies such as nilotinib or dasatinib, imatinib remains widely prescribed in frail or elderly patients with CML. As with most oral targeted therapies, the pharmacokinetics of imatinib, which is extensively metabolized by the hepatic cytochromes CYP3A4 and CYP3A5 and is a substrate of efflux pumps, is characterized by marked inter‐patient variability [2]. Several groups have demonstrated strong exposure‐response (E‐R) relationships with imatinib, and trough levels of approximately 1000 ng/ml are usually associated with an optimal toxicity/efficacy ratio in CML patients, although this target exposure may vary slightly from study to study [3]. As a result, imatinib has been a paradigm drug for the implementation of therapeutic drug monitoring (TDM) in routine patients, both as a means to check patient compliance and to detect patients with potentially inadequate exposure levels [4]. Several reports have shown that individuals with trough levels below the expected target plasma concentration have a lower probability of complete cytogenetic response (CCyR) and major molecular response (MMR) compared to patients with higher plasma exposures [5, 6, 7, 8].

As an oral targeted therapy, imatinib is extensively metabolized in the liver after rapid and almost complete absorption and is excreted in the bile. Consequently, drug–drug interactions and gene polymorphisms affecting either cytochromes P450 or efflux pumps are common causes of inter‐ or intra‐patient variability with imatinib. In population pharmacokinetic modeling, body weight, age, sex, hemoglobin, white blood count (WBC), disease diagnosis and plasma alpha1‐acid glycoprotein have been identified as putative covariates likely to explain variability in imatinib clearance or volume of distribution. However, their influence is considered to be too limited to be used for subsequent dose adjustment [9, 10]. Consequently, there is no a priori adaptive dosing strategy for imatinib, which is given as a 400 mg QD flat dose. Nevertheless, variability in plasma levels could have a detrimental effect on clinical outcomes, as demonstrated by studies showing that poor adherence had a significant impact on MMR [8, 11]. Consequently, TDM‐guided dosing has been shown to significantly improve MMR, i.e. by increasing the imatinib dose in patients with trough levels below the pharmacologically active concentrations [12]. At our institute, TDM is performed with several oral targeted therapies, including imatinib, as part of the routine care of cancer patients. Using a dedicated model, we can simulate trough levels from a single blood sample and adjust dosing to ensure that a trough level of 1000 ng/ml is achieved in an agnostic manner, i.e. irrespective of the causes of inadequate exposure at the start of treatment. There is a paucity of data on the effect that BMI may have on imatinib exposure. Of note, an association has been found between low BMI and adverse events [13], but no PK support was used to determine whether this could be due to plasma overexposure.

Here we investigated the impact of BMI, among other factors related to patient corpulence, on the exposure of imatinib in real‐world adult CML patients treated with standard imatinib. Additionally, how adaptive dosing might limit its impact on clinical outcomes was investigated.

## 2 PATIENTS & METHODS

### 2.1 Patients

This was a retrospective, non‐interventional, observational study conducted at the Department of Hematology, La Conception University Hospital of Marseille (APHM), Marseille, France. After approval by the Review Board of the APHM (study registered as PADSS4XPQ3), routine clinical and biological data, including imatinib serum concentrations, were retrieved from our institution's databases. Other collected data were: standard anthropomorphic data, biological data, clinical data (i.e., efficacy or MMR (i.e., BCR:ABL1/ABL1 ratio on the International Scale [BCR:ABL1^IS^] < 0.1%) at 12 months and adverse events recorded following the CTCAE grading), and when available, information on comedications during imatinib intake. Only severe toxicities (i.e., grade 3 and above) showing during the first 3 months of treatment were considered. Based upon therapeutic drug monitoring performed at steady state, i.e., at least 4 days after treatment initiation, imatinib dosing was likely to be subsequently modified next (see next paragraph: Routine imatinib Therapeutic Drug Monitoring and adaptive dosing). Body weight, height, Body Mass Index, sex, age, and co‐medications were all recorded at baseline. No data on herbal medicine was available in patients' records. For further analysis, patients were divided into four subgroups: BMI ≤ 18.5; 18.5 < BMI ≤  24.9; 25 ≤  BMI ≤  29.9, and BMI ≥ 30, according to the WHO classification [14].

### 2.2 Routine Imatinib therapeutic drug monitoring and adaptive dosing

Imatinib was measured as part of the routine Therapeutic Drug Monitoring program for oral targeted therapies in our institution. Briefly, patients treated with oral targeted therapies are blood‐withdrawn at a steady state, usually 5–10 days after the treatment started. After centrifugation, plasma was isolated, and proteins were precipitated upon acidic condition. Supernatant was next analyzed for imatinib concentration using a simple multiplex LC–MS/MS method fully validated following EMA and ISO15189 standards. Imatinib was assayed over a 25–2000 ng/ml range, with both precision and accuracy <15% and a limit of quantitation of 25 ng/ml. For each patient, imatinib plasma concentrations and the exact timing of the sampling were then implemented in a Shiny interface of the Monolix® software (Lixoft, France). Using standard nonlinear mixed effects modeling and population‐pharmacokinetics (pop‐PK) approach, this allowed simulation of the trough level should the sampling time miss the T24H, and the system was able to make a dose suggestion with respect to a target exposure set as a trough level of 1000 ng/ml for the subsequent administrations. The pop‐PK parameters of imatinib implemented in Monolix® were derived from the ones extensively described in the literature [3, 15], with single‐compartment model, linear elimination and zero‐order absorption, inter‐individual variability on volume of distribution V and total clearance Cl and combined error model, with the following pop‐PK parameters: Cl_pop = 13.8, Tk0_pop = 1.5, V_pop = 252, Ω_Cl = 0.319, Ω _V = 0.314, a = 0.25, b = 0.26. Population modeling allows the time course of imatinib in plasma to be described, considering inter‐individual variability, and also provides the statistical distribution *n* of each PK parameter. By further implementing individual imatinib concentration and sampling time, we could then calculate the conditional distribution according to the statistical law with respect to the measured concentrations [16] Using these individual PK parameters, the software enables to simulate different dosing regimens (i.e. from 100 mg to 1000 mg QD in 100 mg steps) and see the percentage of PK profiles reaching the 1000 ng/ml target Cmin defined by Picard et al [17] This model for adaptive dosing of imatinib was implemented in Monolix® (Lixoft, France) and made available online using a Shiny application (www.lixoft.com). In addition to PK sampling, imatinib dosing could be further adjusted based on clinical considerations.

### 2.3 Statistical analysis

A trough level of 1000 ng/ml was considered the target exposure to be achieved. Regarding the bioanalytical precision (i.e. ± 15%) of the LC–MS/MS method used for the determination of imatinib in plasma, a range of 900–1100 ng/ml was considered acceptable. All statistical analyses and differences between groups were performed using MedCalcV22. 007 (Ostende, Belgium). Univariate analysis was initially performed using standard regression analysis. Further multivariate analysis was performed using the MedCalc® stepwise method. To further investigate the influence of BMI on imatinib exposure in patients divided into three separate groups, additional one‐way ANOVA with Newman Keuls multiple comparisons testing, Chi‐2 tests, and additional Krustal‐Wallis non‐parametric testing with Dunns post‐hoc analysis were performed. BMI was tested both as a categorical and a continuous variable. With respect to the limited number of patients, a *p*‐value of 0.05 was regarded as statistically significant.

## 3 RESULTS

We collected data from 60 adult patients (mean age 56 ± 20 years, range [18–89], M33/27F) starting imatinib (400 mg QD with possible a priori adjustment to 300 mg based on age or clinical considerations such as poor performance status or multiple comorbidities, i.e., elderly patients being always treated with a 300 mg starting dose) for documented CML (Table 1). Consequently, the starting Imatinib dose was 400 mg for 49 patients (82%) and 300 mg for 11 patients (18%).

**TABLE 1:** Patients characteristics.

Patients characteristics (*n* = 60)	
Age years (mean, range)	56, 18–89
Sex (M/F)	33/27
Body Weight kg (mean ±SD)	72.4 ± 17,8
Height cm (mean ±SD)	167 ± 11,2
BSA m^2^ (mean ±SD)	1.82 ± 0,27
BMI (kg/m^2^):	
18,5 (%)	10
18,5 < BMI ≤ 24,9 (%)	32
25 ≤ BMI ≤ 29.9 (%)	41
≥30 (%)	17
Co‐medications (*n* = 35):	
1 (%)	8.5
1 to 5 (%)	42.8
5 to 8 (%)	37.1
>8 (%)	11.4

**FIGURE 1:**
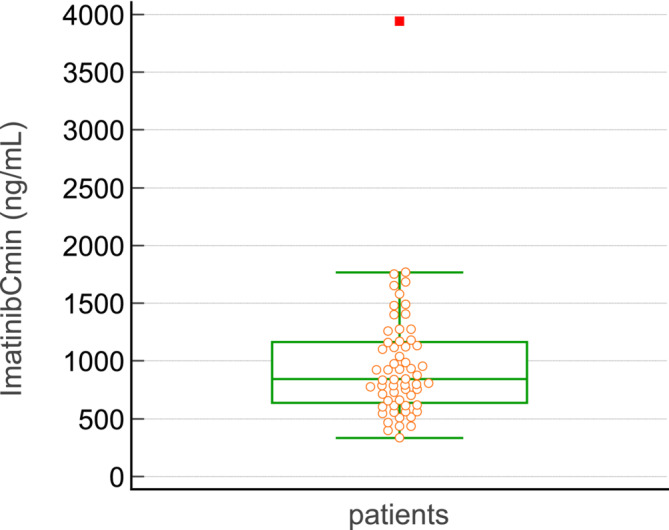
Interpatient variability on imatinib Cmin. Distribution of trough levels of imatinib at steady‐state after treatment initiation. Inter‐patient variability was 56% upon standard dosing.

The mean imatinib dose at baseline was 379 ± 41 mg (median = 400 mg, range 300–400 mg). Mean ±SD were for body weight: 72.4 ± 17.8 kg, height: 167 ± 11.2 cm, body surface area = 1.82 ± 0.27 m^2^, BMI = 25.7 ± 5.3 kg/m^2^ (range: 17–39.2). Co‐medication was described in detail in only 35 patients (58%). Of these, 22 patients (63%) were taking at least one drug with potential inhibitory or inducing properties that could cause a drug–drug interaction (DDI) with imatinib. When available, the number of concomitant drugs patients were taking was: 1 (8%), between 2 and 5 (43%), between 6 and 8 (37%), and >8 drugs (11%). Trough imatinib levels were 994.2 ± 560.6 ng/ml (CV: 56%) range: 334–3944, median: 844 ng/ml) at steady state at the start of treatment (Figure 1).

The observed Cmin values did not follow a normal distribution (W = 0.7492, *p* < 0.001, Shapiro–Wilk test). When weighted by dose (i.e. Ctrough/dose), the inter‐patient variability was still 67%. Overall, 43% of patients were below target at baseline and 28% were above target. Only 29% of patients were in the target range.

The search for correlations between the collected anthropomorphic parameters and baseline trough levels was performed by univariate and then stepwise multivariate analysis, as shown in Table 2. Patients' BMI was tested both as a categorical variable (i.e., <18.5, 18.5–24.9, 25–29.9, and ≥30 kg/m^2^) and as a continuous variable.

**TABLE 2:** Univariate and multivariate (stepwise mode) regression analysis of covariates impacting imatinib trough levels at treatment initiation.

	Univariate	Multivariate
Baseline parameter	*p*	*p*
Age	**0.032**	**0.004**
Sex	>0,05	‐
Dose	>0,05	‐
Weight	**0.0176**	>0,05
Height	**0.0409**	>0,05
Body surface area	**0.0098**	>0,05
Body mass index	**0.0184**	**0.024**
Co‐medication	>0.05	‐

On univariate analysis, age and all covariates associated with corpulence (i.e., weight, height, BSA, BMI) were associated with trough levels (*p* < 0.05). Conversely, sex and concomitant medication were not associated with imatinib Cmin. However, after multivariate analysis, only BMI (*p* = 0.018) and age (*p* = 0.0024) remained associated with plasma exposure (*F* ratio = 8.6, *p* = 0.0011). When further multivariate analysis was performed with BMI now considered as a continuous variable, BMI was still associated with trough levels (*p* = 0.0024, *F* ratio = 9.6044 [*p* = 0.0006]).

**FIGURE 2:**
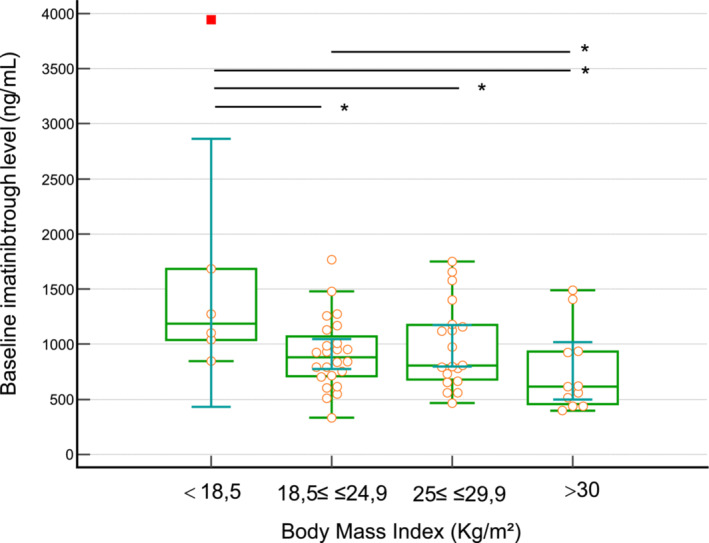
Differences in baseline imatinib exposure depending on BMI. Comparison in Imatinib trough levels depending on patient's BMI. Patients with BMI < 18.5 had mean Imatinib Cmin higher than the rest of the population, *: *p* < 0.05, Krustal‐Wallis non‐parametric test with Dunns post‐hoc analysis.

When comparing exposure levels according to BMI, trough concentrations at baseline were 1572 ± 1341 ng/ml, 973 ± 333 ng/ml, and 890 ± 349 ng/ml in patients with BMI < 18.5, BMI between 18.5 and 24.9 and BMI ≥ 25, respectively (Figure 2). Focusing on the subset of patients with BMI≥ 30, the median Cmin was 616 ng/ml, but the mean was 758 ± 437 ng/ml, because two patients had trough values above 1400 ng/ml, which strongly influenced the mean values recorded in these patients. Exposure in patients with BMI < 18.5 was statistically higher than in the other three groups (*p* < 0.05, ANOVA with Student's Newman–Keuls multiple comparison test). Using further Tukey–Kramer or Scheffe post‐hoc tests, trough values remained statistically higher in patients with a BMI < 18.5 than in those with a BMI > 24.9 (*p* < 0.05). When further using Krustal Wallis non‐parametric testing, trough levels remained significantly different depending on BMI (*p* = 0.02) with patients with BMI < 18.5 exhibiting statistically significant higher concentrations than other patients (Dunns or Conover post‐hoc tests, *p* < 0.05).

### 3.1 PK‐guided dose adjustment

**FIGURE 3:**
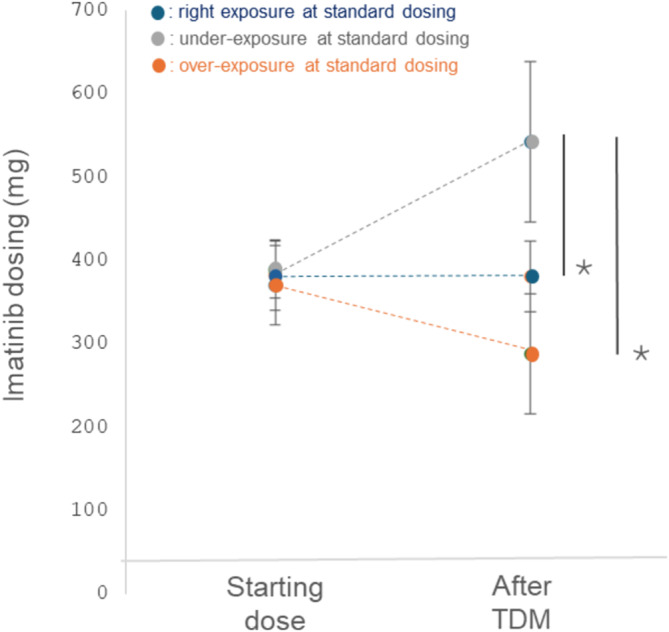
Differences in imatinib dosing before and after TDM. PK‐guided dosing of Imatinib. Depending on exposure measured at standard dosing (mean: 379 mg imatinib), either decrease (mean: 287.5 mg) or increase (mean: 536 mg) in dosing was undertaken next (* *p* < 0.001, ANOVA with Newman Keuls multiple comparison testing).

A total of 74% patients had their imatinib dosing changed based upon TDM and PK‐guided dosing: 33% had a decrease and 41% an increase in dosing. Patients with decreased dosing had a mean imatinib trough levels at baseline of 1482 ng/ml (range: 1039–3944) whereas patients with increased dosing had a mean imatinib trough level of 636.5 ng/ml (range: 334–799). The remaining 26% had their dosing unchanged (i.e., 300 or 400 mg depending on the starting dose). There was no statistical difference in dosing among patients at treatment initiation (*p* = 0.411, One‐Way ANOVA). However, after TDM and PK‐guided dosing, patients below the target threshold had dosing increased to an average 536 mg (range: 400–700), patients in the target threshold had no change in dosing (mean: 378 mg, range 300–400) whereas patients above the target threshold had a decrease in dosing (mean: 287.5 mg, range 200–400). This difference in dosing was statistically significant (*p* < 0.001, One‐Way ANOVA with Newman Keuls multiple comparison testing). Figure 3 displays the change in dosing depending on the initial exposure measured in patients.

Regarding changes in imatinib dosing depending on BMI status, no statistical difference was observed when treatment started (mean Imatinib dose: 380 ± 44, 363 ± 49, 400 ± 0, and 371 ± 48 mg in patients with BMI < 18.5, comprised between 18.5 and 24.9, comprised between 25 and 29.9, and ≥30 kg/m^2^, respectively, *p* = 0.580 ANOVA). However, after TDM, a statistical difference was observed between mean dosing (333 ± 114, 400 ± 49, 428 ± 160,and 440 ± 118 in patients with BMI < 18.5, comprised between 18.5 and 24.9, comprised between 25 and 29.9, and ≥30, respectively, *p* = 0.05 ANOVA with Newman Keuls multiple comparison testing).

### 3.2 Clinical outcome

Major Molecular Response was observed in 80% of patients, and 31.6% of patients experienced at least one or more severe toxicities during the 3‐month evaluation period. There was no statistical difference in efficacy between patients according to BMI (*p* = 0.33, Chi‐2 test). Similarly, no difference in adverse events was observed (*p* = 0.911, Chi‐2 test), despite the marked differences in exposure observed at baseline. After dose adjustment, no difference was observed depending on the final dosing (i.e., decreased, increased or maintained) in efficacy (*p* = 0.098, chi‐2 test) or in adverse events (*p* = 0.157, chi‐2 test).

## 4 DISCUSSION

Implementing precision medicine at the bedside is critical in oncology and developing adaptive dosing strategies in patients should help maintaining drug exposure within the therapeutic window. There is already abundant literature describing E‐R relationships with imatinib in CML patients and several other studies have already demonstrated how implementing PK‐guided dosing could remarkably improve clinical outcomes [12]. In this work, we have studied to what extent BMI could have an impact on imatinib PK. Many papers have already investigated the possible causes of inter‐patient variability in pharmacokinetics. To date, body weight, ideal body weight, and lean mass have all been associated with PK parameters of imatinib, although their impact remains sometimes modest [18]. In a retrospective study, elevated body weight was associated with decreased exposure levels and lack of efficacy [19]. Of note, others have failed to find a significant relationship between age, height, body weight, or BSA and imatinib plasma levels [20], highlighting the fact that confounding factors such as comorbidities, poor adherence, or drug–drug‐interactions (DDI) are probably at play too. Surprisingly here, co‐medications were not associated with imatinib exposure, most probably because upfront pharmaceutical mediations prevented major DDI and helped avoid strong CYP450 inducers or inhibitors. In addition, comprehensive information regarding co‐medications, including over‐the‐counter medications, was available in about half of the patients only, leading to a lack of statistical power to be conclusive. Consequently, the fact that here, co‐medications were found to have no significant impact does not necessarily mean that they do not play a major role in the PK variability of oral targeted therapies [21]. It has to be stressed out that although 1000 ng/ml is usually considered as the target trough level for Major Molecular Response, other studies have suggested that a wider target‐range (i.e., 1000–1500 ng/ml) could be considered for CML patients because concentrations above 1000 ng/ml were not necessarily associated with more severe toxicities [22]. However, others have suggested that even low‐grade adverse events should be avoided at all costs because of their potential impact on adherence, pill fatigue, and even chronicity of low‐grade toxicities with imatinib [23], which explains why some prescribers prefer to maintain 1000 ng/ml as the target exposure. Here, we observed a marked inter‐patient variability in imatinib trough levels at treatment initiation with standard dosing (i.e., CV > 50%), an observation fully in line with what was observed in previous clinical reports [10, 15]. The fact that some patients were treated with 300 mg and not 400 mg does not explain this observed variability, since when considering differences in starting dose, the inter‐individual variability was even higher. Consequently, only 29% patients were in the therapeutic window when treatment started, an observation in line with other clinical reports showing that less than 30% patients have plasma levels in the range of the expected therapeutic window [24]. Our data have shown that trough levels upon treatment initiation are related to the BMI upon multivariate analysis and that patients with BMI ≤ 18.5 had significantly higher (i.e., about +70%) plasma concentration as compared with other patients. Conversely, patients with higher BMI tend to have trough levels lower than other individuals, although this difference was not found to be significant. Of note, when focusing on the patients with extreme BMI (i.e., >30), one would have expected very low concentrations but the Cmin was 758 ± 437 ng/ml, only 23% below the average imatinib trough levels, due to two outliers patients in this subset with unexpected high imatinib levels despite Class I obesity. This illustrates how the small number of patients monitored in this single‐institute study limits some of the interpretations. However, here our data comparing baseline trough levels as a function of BMI suggest that special attention should be paid to patients with extreme BMI (i.e., ≤18.5 or ≥ 30 kg/m^2^), as they deviate symmetrically from the target exposure of 1000 ng/ml. Overall, PK‐guided dosing allowed patients with too low or too high plasma levels to have their dose either increased or decreased before a poor clinical outcome (i.e. severe toxicities or lack of efficacy) was observed. Following PK‐guided dosing, no difference in efficacy or toxicity was observed eventually. Only severe toxicities during the first three months were considered because patients were outpatients, less serious toxicities were not always correctly monitored in their records, and hospital visits were less frequent after this time, so later toxicities were more likely to be misreported or missed. It has to be highlighted that other parameters such as hemoglobin or white blood cells were not tested here, because previous reports failed to identify them as being strongly associated with imatinib PK variability [9]. Conversely, alpha‐(1) glycoprotein acid was not tested either, despite being strongly associated with imatinib PK [10], because orosomucoid is not routinely measured in routine settings, and as a real‐world study, this parameter was not available in our patients. Taken together, our data suggest that BMI may be an important covariate associated with imatinib pharmacokinetics and that patients with low BMI have higher exposure levels with a potentially greater risk of severe toxicities if adaptive dosing is not used next. Indeed, imatinib has been shown to be toxic to muscle tissue and to increase creatine kinase levels [25, 26]. Our data may explain the association between low BMI and musculoskeletal pain previously found by Katagiri et al, but without a clear mechanistic explanation thus far [13]. Our data suggest that pharmacometric models using relevant covariates such as BMI could therefore be useful to anticipate such safety concerns, although previous POP‐PK studies failed to implement such characteristics, including BMI, in their final models [18]. In addition, our data suggest also that patients with sudden changes in their BMI (e.g., after bariatric surgery or sleeve) should have their dosing re‐appraised, since, in addition to directly affecting the oral bioavailability of oral targeted therapies [27, 28], it could further change the exposure levels of imatinib due to subsequent changes in BMI. Of note, a methodological weakness of this study is the small number of patients from a single institution, resulting in, for example, too few patients with BMI > 30 to be properly analyzed separately ‐ however, we believe that its “real world” nature and the fact that no selection criteria were set as a prerequisite make it interesting, in line with the pragmatic clinical trials defined by the EMA and the EORTC [29]. Another weakness is the fact that at our institution, TDM is only required at the start of treatment and is not carried out longitudinally after dose adjustment. However, the clinical data and the lack of difference in efficacy and toxicity endpoints suggest that the marked variability in exposure at the start of treatment was adequately smoothed by the adaptive dosing strategy implemented after TDM. Finally, the large inter‐individual variability in imatinib exposure observed in this real‐world study (i.e., >50%) suggests that beyond imatinib, addressing the issue of inter‐patient variability and developing TDM with other oral targeted therapies that share the same PK profile for the treatment of leukemia patients (i.e., nilotinib, dasatinib, bosutinib, ponatinib, and aciminib) may also be clinically relevant because they share similar PK patterns.

## 5 CONCLUSION

Our data suggest that patients with a low BMI should have their starting dose reduced (i.e., 300 mg), as patients with trough levels above the target threshold in our study were successfully treated with a mean dose of 287 mg of imatinib (range: 200–400 mg) without compromising efficacy at 12 months. Conversely, increasing the dose to a mean of 536 mg (range: 400–700 mg) in patients below the target Cmin did not increase the incidence of severe toxicities which were equally distributed among the patients. This suggests that patients with a BMI ≥ 30 kg/m^2^ could safely have their imatinib dose increased, as these patients had trough levels below the target exposure on standard dosing. Our results therefore show that routine TDM with an adaptive dosing strategy helps to smooth out the variability in pharmacodynamic effects, despite the marked variability in pharmacokinetics when treatment is started at standard dose, partly due to differences in measured BMI. Indeed, no difference in efficacy or tolerability was observed in our patients, regardless of their BMI status, after this dosing was personalized. Should TDM not be feasible with subsequent PK‐guided dosing, our results call for special attention to be paid to patients with low BMI who are to be treated with imatinib, as they may be overexposed, and to patients with high BMI, as they may be underexposed. As the variability affecting imatinib is multifactorial, pharmacist mediation with trained staff could help to guide upfront adaptive dosing if TDM is not available.

## AUTHOR CONTRIBUTIONS

P Maroselli: collected data, analyzed data, wrote the MS. R Fanciullino: collected data, analyzed data, wrote the MS. L Farnault: collected data J.Colle: collected data; P Roche: collected data, G Venton: collected data; R Costello: collected data; J.Ciccolini: collected data, analyzed data, wrote the MS. All authors: read and approved the final manuscript.

## CONFLICT OF INTEREST STATEMENT

The authors have no conflict of interest to disclose en relation with the present work.

## PERMISSION TO REPRODUCE MATERIAL FROM OTHER SOURCES

Not applicable.

## ETHICS APPROVAL

The retrospective data collection and statistical analysis were approved by our Review Board (Cellule d'Evaluation at the Direction de la Recherche Clinique & de l'Innovation [DRCI], APHM) and followed the European recommendations regarding the Clinical Trials Regulation (EU) 536/20141 and the General Data Protection Regulation (GDPR; EU) 2016/6792.

## PATIENT CONSENT

With respect to the non‐interventional, retrospective nature of this study, Written Informed consent was waived by the Assistance Publique Hôpitaux de Marseille Review Board.

## CLINICAL TRIAL REGISTRATION

This study was granted the French MR004 legal status for non‐interventional studies and registered as such as #PADSA3GKW7.

## Data Availability

All raw data (i.e., PK metrics, anthropomorphic and clinical data) will be provided upon request to the corresponding author.

## References

[1] Hochhaus A , Larson RA , Guilhot F , et al. Long‐term outcomes of imatinib treatment for chronic myeloid leukemia. N Engl J Med. 2017;376:917‐992. 28273028 10.1056/NEJMoa1609324 PMC5901965

[2] Peng B , Lloyd P , Schran H . Clinical pharmacokinetics of imatinib. Clin Pharmacokinet. 2005;44(9):879‐894. doi:10.2165/00003088-200544090-00001 16122278

[3] Gotta V , Buclin T , Csajka C , Widmer N . Systematic review of population pharmacokinetic analyses of imatinib and relationships with treatment outcomes. Ther Drug Monit. 2013;35(2):150‐167. doi:10.1097/FTD.0b013e318284ef11 23503441

[4] Gotta V , Widmer N , Montemurro M , et al. Therapeutic drug monitoring of imatinib: Bayesian and alternative methods to predict trough levels. Clin Pharmacokinet. 2012;51(3):187‐201. doi:10.2165/11596990-000000000-00000 22339450

[5] Adattini JA , Adiwidjaja J , Gross AS , McLachlan AJ . Application of physiologically based pharmacokinetic modeling to understand real‐world outcomes in patients receiving imatinib for chronic myeloid leukemia. Pharmacol Res Perspect. 2023;11:e01082. 37417254 10.1002/prp2.1082 PMC10326685

[6] Gotta V , Bouchet S , Widmer N , et al. Large‐scale imatinib dose–concentration–effect study in CML patients under routine care conditions. Leuk Res. 2014;38(7):764‐772. doi:10.1016/j.leukres.2014.03.023 24844604

[7] Bouchet S , Titier K , Moore N , et al. Therapeutic drug monitoring of imatinib in chronic myeloid leukemia: experience from 1216 patients at a centralized laboratory. FundamClinPharmacol. 2013;27(6):690‐697. doi:10.1111/fcp.12007 23113675

[8] Larson RA , Druker BJ , Guilhot F , et al. Imatinib pharmacokinetics and its correlation with response and safety in chronic‐phase chronic myeloid leukemia: a subanalysis of the IRIS study. Blood. 2008;111(8):4022‐4028. doi:10.1182/blood-2007-10-116475 18256322

[9] Schmidli H , Peng B , Riviere GJ , et al. Population pharmacokinetics of imatinib mesylate in patients with chronic‐phase chronic myeloid leukaemia: results of a phase III study. Br J ClinPharmacol. 60:35‐44. 10.1111/j.1365-2125.2005.02372.x PMC1884912 15963092

[10] Widmer N , Decosterd LA , Csajka C , et al. Population pharmacokinetics of imatinib and the role of alpha‐acid glycoprotein. Br J ClinPharmacol. 2006;62:97‐112. 10.1111/j.1365-2125.2006.02719.x PMC1885072 16842382

[11] Ibrahim AR , Eliasson L , Apperley JF , et al. Poor adherence is the main reason for loss of CCyR and imatinib failure for chronic myeloid leukemia patients on long‐term therapy. Blood. 2011;117:3733‐3736. 21346253 10.1182/blood-2010-10-309807 PMC6143152

[12] Johnson‐Ansah H , Maneglier B , Huguet F , et al. Imatinib optimized therapy improves major molecular response rates in patients with chronic myeloid leukemia. Pharmaceutics. 2022;14(8):1676. doi:10.3390/pharmaceutics14081676 36015302 PMC9414005

[13] Katagiri S , Tauchi T , Ando K , Okabe S , Gotoh M , Ohyashiki K . Low body weight and body mass index may be associated with musculoskeletal pain following imatinib discontinuation in chronic myeloid leukemia. Leuk Res Rep. 2017;7:33‐35. 28462083 10.1016/j.lrr.2017.04.002 PMC5402624

[14] https://www.who.int/data/gho/data/themes/topics/topic-details/GHO/body-mass-index Last Access 6th Nov 2024.

[15] Delbaldo C , Chatelut E , Ré M , et al. Pharmacokinetic‐pharmacodynamic relationships of imatinib and its main metabolite in patients with advanced gastrointestinal stromal tumors. Clin Cancer Res. 2006;12(20):6073‐6078. doi:10.1158/1078-0432.CCR-05-2596 17062683

[16] Lavielle M , Ribba B . Enhanced method for diagnosing pharmacometric models: random sampling from conditional distributions. Pharm Res. 2016;33(12):979‐2988. doi:10.1007/s11095-016-2020-3 27604892

[17] Picard S , Titier K , Etienne G , et al. Trough imatinib plasma levels are associated with both cytogenetic and molecular responses to standard‐dose imatinib in chronic myeloid leukemia. Blood. 2007;109:3496‐3499. 17192396 10.1182/blood-2006-07-036012

[18] Chien YH , Würthwein G , Zubiaur P , et al. Population pharmacokinetic modelling of imatinib in healthy subjects receiving a single dose of 400 mg. Cancer Chemother Pharmacol. 2022;90(2):125‐136. doi:10.1007/s00280-022-04454-y 35831644 PMC9360108

[19] Van Obbergh F , Knoops L , Devos T , et al. The clinical relevance of imatinib plasma trough concentrations in chronic myeloid leukemia. A Belgian study. Clin Biochem. 2017;50(7‐8):452‐454. doi:10.1016/j.clinbiochem.2016.12.006 28017570

[20] Escudero‐Ortiz V , Domínguez‐Leñero V , Catalán‐Latorre A , Rebollo‐Liceaga J , Sureda M . Relevance of therapeutic drug monitoring of tyrosine kinase inhibitors in routine clinical practice: a pilot study. Pharmaceutics. 2022;14(6):1216. doi:10.3390/pharmaceutics14061216 35745789 PMC9228468

[21] Ferrer F , Fanciullino R , Milano G , Ciccolini J . Towards rational cancer therapeutics: optimizing dosing, delivery, scheduling, and combinations. Clin Pharmacol Ther. 2020;108(3):458‐470. doi:10.1002/cpt.1954 32557660

[22] García‐Ferrer M , Wojnicz A , Mejía G , Koller D , Zubiaur P , Abad‐Santos F . Utility of therapeutic drug monitoring of imatinib, nilotinib, and dasatinib in chronic myeloid leukemia: a systematic review and meta‐analysis. Clin Ther. 2019;41(12):2558‐2570.e7. doi:10.1016/j.clinthera.2019.10.009 31812340

[23] Jabbour EJ , Kantarjian H , Eliasson L , Cornelison AM , Marin D . Patient adherence to tyrosine kinase inhibitor therapy in chronic myeloid leukemia. Am J Hematol. 2012;87(7):687‐691. doi:10.1002/ajh.23180 22473898 PMC11726351

[24] Lankheet NA , Knapen LM , Schellens JH , Beijnen JH , Steeghs N , Huitema AD . Plasma concentrations of tyrosine kinase inhibitors imatinib, erlotinib, and sunitinib in routine clinical outpatient cancer care. Ther Drug Monit. 2017;36:326‐334. 10.1097/FTD.0000000000000004 24305627

[25] Gordon JK , Magid SK , Maki RG , Fleisher M , Berman E . Elevations of creatine kinase in patients treated with imatinib mesylate (Gleevec). Leuk Res. 2010;34(6):827‐829. doi:10.1016/j.leukres.2009.11.002 19963273

[26] Omran MM , Ibrahim AB , Abdelfattah R , Shouman SA , Hamza MS . Imatinib pharmacokinetics and creatine kinase levels in chronic myeloid leukemia patients: implications for therapeutic response and monitoring. Eur J Clin Pharmacol. 2024;80(7):1061‐1068. doi:10.1007/s00228-024-03675-9 38536418 PMC11156749

[27] Haddad FG , Kantarjian HM , Bidikian A , et al. Association between bariatric surgery and outcomes in chronic myeloid leukemia. Cancer. 2023;129(12):1866‐1872. doi:10.1002/cncr.34725 36882573

[28] Lau C , van Kesteren C , Cao YX , et al. Varying concentrations of tyrosine kinase inhibitors in chronic myeloid leukemia patients following bariatric surgery: a case series. Ann Hematol. 2024;103(11):4765‐4771. doi:10.1007/s00277-024-05924-4 39129028 PMC11534994

[29] https://www.ema.europa.eu/en/documents/presentation/presentation-pragmatic-clinical-trials-preliminary-shared-experience-under-ctr-d-lacombe-eortc_en.pdf Last Access 6th Nov 2024.

